# The Case for Telemedical Early Medical Abortion in England: Dispelling Adult Safeguarding Concerns

**DOI:** 10.1007/s10728-021-00439-9

**Published:** 2021-10-23

**Authors:** Jordan A. Parsons, Elizabeth Chloe Romanis

**Affiliations:** 1 Bristol Medical School, University of Bristol, Bristol, UK; 2 Durham Law School, Durham University, Durham, UK

**Keywords:** Reproductive health, Early medical abortion, Telemedicine, Safeguarding, Health policy

## Abstract

Access to abortion care has been hugely affected by the COVID-19 pandemic. This has prompted several governments to permit the use of telemedicine for fully remote care pathways, thereby ensuring pregnant people are still able to access services. One such government is that of England, where these new care pathways have been publicly scrutinised. Those opposed to telemedical early medical abortion care have raised myriad concerns, though they largely centre on matters of patient safeguarding. It is argued that healthcare professionals cannot adequately carry out their safeguarding duties if the patient is not in the room with them. These concerns lack empirical support. Emerging evidence suggests that safeguarding processes may, in fact, be more effective within telemedical abortion care pathways. In this article, we address two specific safeguarding concerns: (1) that a remote consultation prevents a healthcare professional from identifying instances of abuse, and (2) that healthcare professionals cannot reliably confirm the absence of coercion during a remote consultation. We demonstrate that such concerns are misplaced, and that safeguarding may actually be improved in telemedical care pathways as victims of abuse may find it easier to engage with services. It is inevitable that some individuals will fall through the net, but this is unavoidable even with in-person care and thus does not constitute a strong critique of the use of telemedicine in abortion care. These safeguarding concerns set aside, then, we argue that the current approval that enables telemedical early medical abortion should be afforded permanence.

## Introduction

In March 2020, the governments of England [21], Wales [66], and Scotland [61] issued approval orders—under powers afforded by the Abortion Act 1967 (AA 1967)^1^—to permit home use of mifepristone for the purposes of early medical abortion (EMA). The orders permitted the use of telemedicine in the delivery of care (TEMA; telemedical early medical abortion). This change was made in response to the circumstances created by the COVID-19 pandemic. Measures to prevent the spread of the virus made the delivery of in-person abortion care more challenging, introducing yet more access barriers in an area of healthcare that was already inaccessible to many, which forced government action.^2^

Since TEMA has been made temporarily lawful in Great Britain, data have emerged to show the benefits of the service: accessibility, patient satisfaction [41], and safety [3]. Whilst the use of telemedicine to provide abortion care was by no means novel, having been implemented in other countries more than a decade earlier [33], the approval orders were publicly scrutinised by politicians and anti-abortion campaign groups for, among other concerns, eroding the safeguarding abilities of care providers. Such concerns have also been raised by some medical professionals. Opponents have argued that the absence of a remote consultation precludes the provider from effectively identifying instances of abuse or neglect, thereby preventing them from executing their duty of care. If true, this would interfere with professionals’ safeguarding duties under the Care Act 2014 and result in potentially avoidable harm to victims of abuse and neglect.

These concerns, we argue, are misplaced. Properly implemented telemedicine enables safeguarding to be carried out effectively, with some evidence even suggesting it can be improved in the context of TEMA. In this article, we first outline abortion law in England (and Wales) as it pertains to TEMA. Our discussion then focuses on the safeguarding concerns raised and how a TEMA care pathway can appropriately map onto the requirements of both the Care Act 2014 and general professional guidance and expectations. We demonstrate that the evidence does not align with two key safeguarding concerns raised by opponents of TEMA: (1) that a remote consultation prevents a healthcare professional from identifying instances of abuse, and (2) that healthcare professionals cannot reliably confirm the absence of coercion during a remote consultation.

Our focus in this paper is on England, with the Care Act 2014 as central to our discussion.^3^ However, the principles that ground the concept of safeguarding—and, by extension, our discussion—should be thought of as more widely applicable, extending to Scotland, Wales, Northern Ireland, and beyond the UK [57]. The straightforward conclusion that vulnerable patients accessing TEMA can be identified and helped in much the same way as when care is delivered in person is not unique to the context of the Care Act 2014, and we use the Act simply as a tool for exploration. We also limit our discussion to adult safeguarding but recognise that many of our conclusions could also apply to people under 18 (with capacity) seeking access to abortion. Further, we focus exclusively on adults with capacity.

This discussion of the adequacy of safeguarding in the TEMA context is important at a time when the governments of Great Britain are considering whether to make TEMA services permanently lawful, particularly given that opponents base their opposition largely on safeguarding concerns.

## TEMA and Safeguarding Concerns

### Abortion Law in England (and Wales)

All termination of pregnancy in England (and Wales) remains a criminal offence under the Offences Against the Person Act 1861^4^ and the Infant Life (Preservation) Act 1929.^5^ Abortion is, however, lawful when provided by a registered medical practitioner in circumstances outlined in the AA 1967.^6^

The abortion must be provided on one of the (socio-)medical grounds detailed in Section 1(1) of the AA 1967. The first ground—that the risk associated with termination is less than the risk associated with continuing the pregnancy^7^—is so broadly construed that it renders all pregnancies legally terminable within the time period that medical abortion is a treatment option. In addition to satisfying one of the grounds for abortion, the treatment must be provided in line with further conditions: the ground for provision must be certified by two registered medical practitioners,^8^ the abortion must be carried out by (meaning supervised by (paras 569–570) [58]) a medical practitioner in a place specifically approved for the provision of abortion,^9^ and treatment must be provided within specified gestational limits.^10^ Prior to the changes made in 2020, misoprostol (the second EMA drug) was approved for use in a patient’s home—defined as the place of their usual residence in England [23]—but mifepristone (the first EMA drug) could only be administered in a clinic. This prohibited the establishment of a fully^11^ telemedical abortion service in England.

The situation prior to the introduction of TEMA was in many ways problematic. First, the impermissibility of remote prescription of EMA medications was at odds with the policy of regulators in other healthcare contexts [4]. The Medicines Act 1968 requires that ‘prescription only medicines’—which includes both mifepristone and misoprostol—must be supplied only per prescription by an appropriate practitioner, and then administered in line with that practitioner’s directions. There is no specific requirement that the practitioner examine the patient to issue a prescription, and there is widespread acceptance that prescription is, in some circumstances, appropriately done without examination or face-to-face consultation. Thus, that the law placed additional requirements on abortion medications interfered with the clinical discretion recognised as important in the context of other medications [4]. Certainly since the outbreak of the COVID-19 pandemic there has been a global embrace of telemedicine, but even beforehand the General Medical Council (GMC) considered remote consultation and prescription appropriate where:

- ‘The patient’s clinical need or treatment request is straightforward
- You can give patients all the information they want and need about treatment options by phone, internet, or video link
- You have a safe system in place to prescribe
- You have access to the patient’s medical records
- You don’t need to examine the patient
- The patient has capacity to decide about treatment’ [29]

Pre-TEMA abortion care also, and somewhat relatedly, raised concerns in terms of access. Abortion clinics in England are concentrated in larger metropolitan areas and/or they serve large geographical areas [10, 42, 45]. As such, people must often travel considerable distances to access their nearest clinic. This can be difficult for people in rural areas, people without access to personal/private transport, people who are socio-economically disadvantaged/with limited income, disabled people, and people working/with existing caring responsibilities as they may find it hard to travel—especially where this is a significant distance [56]. This can often delay people accessing treatment as they take time to make such arrangements [50]. Some people facing these barriers—amongst others—have reported considering travelling to an abortion clinic to be impossible, and so they opted to obtain abortifacients unlawfully over the internet rather than using (inaccessible) formal channels [2].

The problem of access also results in issues around safety, effectiveness, and acceptability of care. On all three counts, where a patient is going to undergo an EMA it is better to do so as soon as possible. As the gestational age increases, the likelihood of incomplete abortion gradually increases [7, 13]. Whilst they remain at a clinically acceptable level within the period such treatment is permitted, they can cause an unnecessarily unpleasant experience for the patient. Hence, delays in accessing care can be problematic. Should the delay be significant enough, it may even result in the patient no longer having the option of EMA by virtue of legal stipulations, resulting in a choice between a surgical abortion or continuing the unwanted pregnancy. Where abortion care is going to be accessed, then, there is good reason to enable swift access. Furthermore, whilst the evidence is clear that self-managed abortion with unlawfully-sourced abortifacients (from recognised providers, such as Women on Web) is safe [30], it is preferable that where people have access to formal channels that these are made available to them—to ensure that their safety is preserved by ensuring they do not face any legal consequences [50], and to ensure that they feel comfortable accessing appropriate medical support in the rare event of complications.

### Understanding TEMA

The use of telemedicine in abortion care is a prominent development in reproductive health. It can be utilised at various stages in a care pathway, including the initial stages of counselling and consultation, the provision of abortifacient medications, advice and support throughout the procedure itself, and for aftercare. Different service providers have embraced telemedicine for different combinations of these elements [50]—it should be noted that those we discuss here are simply a selection and not an exhaustive list of TEMA services.

A Planned Parenthood affiliate in Iowa in the US—Planned Parenthood of the Heartland—introduced the first regulated TEMA service in 2008 [33, 34].^12^ Using this service, patients can attend their nearest clinic to access abortion care even if it does not have an on-site doctor. A patient will attend their nearest clinic for an ultrasound scan and to have their medical history taken. Still in the clinic, they will join a video call with an off-site doctor^13^ who will conduct a remote consultation and, if appropriate, prescribe the drugs immediately^14^—the mifepristone is taken whilst still on the video call, and the patient can take the misoprostol home to complete their treatment [33]. A follow-up appointment is then scheduled to take place within a fortnight, at which point an ultrasound scan is conducted to assess abortion completeness.

More recent has been the move to direct-to-patient dispensing, removing the requirement to attend an abortion clinic. The TelAbortion Project^15^ in the US has, since 2016, allowed patients in certain states^16^ to obtain screening tests at local laboratories and radiology offices before videoconferencing with one of the Study’s abortion provider partners [53, 63]. Eligible patients are then sent mifepristone and misoprostol by post or, if the particular provider is subject to additional restrictions on the prescription of mifepristone, they are made available to collect from a pharmacy. Again, a follow-up is scheduled for testing to determine abortion completeness. Gynuity Health Projects—the organisation that operates the TelAbortion Project—established a secondary study in response to the COVID-19 pandemic. To reduce in-person contact, ultrasound scans were not carried out where this was considered to protect patient welfare—thereby making the service fully remote for some patients—and this was justified by way of the US Food and Drug Administration’s pandemic research guidelines [65]. However, following legal proceedings concerning the prescription of mifepristone [26], this second study was forced to cease operating.

An altogether different service is operated by Women on Web. This model is similar to that of TelAbortion in that it takes a direct-to-patient approach to dispensing abortifacients but differs in the lack of required screening tests and follow-up. It is a fully remote, online service, operating internationally to serve those living in countries where they are ‘unable to access an abortion safely’ [67]. Patients complete an online questionnaire as a consultation. To ascertain gestational age, this questionnaire encourages patients to undergo a pregnancy test or ultrasound scan but does also permit estimation based on last menstrual period.^17^ The patient’s information is then reviewed by a doctor and, if appropriate, the drugs are posted to the patient with instructions and information. This service is unique in that the consultation is asynchronous, enabling the patient to complete it at a time that is most convenient. Incidentally, Women on Web has been the subject of several studies exploring the safety, effectiveness, and acceptability of TEMA [1, 30–32, 40]—this will be discussed further below.

A final model to highlight—and one that is more close to home in terms of our discussion—is the British Pregnancy Advisory Service’s ‘Pills by Post’ service [9]. Pills by Post combines an entirely remote service with synchronous patient-provider communication. An initial consultation (which may be a phone or video call) with a nurse or midwife covers treatment options, medical history, estimating gestational age, whether an ultrasound is indicated, and, in relation to safeguarding matters, an ‘assessment of safety at home’ (p. 4) [9]. This information is then reviewed by two doctors who, if satisfied, prescribe the medication, which is then posted to the patient along with codeine to manage pain and a pregnancy test to confirm treatment success [9]. Largely similar services have been established by other providers in Great Britain, such as the National Unplanned Pregnancy Advisory Service [44] and MSI Reproductive Choices [43], since the temporary change to regulations.

Whilst our position is that a comprehensive TEMA service that wants to embrace the myriad benefits of telemedicine^18^ would incorporate (whilst keeping optional) remote provision of all stages of a care pathway, requiring in-person care only when indicated,^19^ it remains that variation in regulatory frameworks necessitates variation in the application of telemedicine to care pathways. Indeed, TEMA services can very much be considered as existing on a continuum inclusive of ‘partial’ and ‘full’ telemedicine examples [50].

There is a strong body of evidence to demonstrate that TEMA is safe, effective, and acceptable to patients. A systematic review of thirteen studies^20^ found rates of ‘complete abortion, continuing pregnancy, hospitalization, and blood transfusion’ following TEMA up to ten weeks’ gestation to be similar to those reported following in-clinic provision (p. 1094) [25]. Whilst the number of surgical evacuations was slightly higher than expected, there was a high level of acceptability for both patients and providers [25]. The studies included in this review are based primarily on self-reported data, though this does at least reliably indicate high levels of acceptability.

Endler and colleagues’ systematic review was conducted prior to the COVID-19 pandemic. As such, there is now a significant body of more recent research to corroborate their findings [3, 14, 39, 41]. Of particular relevance to our discussion is a study conducted across the three main abortion providers in England to examine the safety, effectiveness, and acceptability of their TEMA care pathways [3]. The study compared TEMA services with traditional, in-person services, finding comparable results on treatment success, serious adverse events, and incidence of ectopic pregnancy [3]. TEMA services saw higher effectiveness (p. 1470) and were highly acceptable to patients (p. 1469) [3].

### Approving TEMA in England

The approval order that enabled TEMA in England was issued on 30 March 2020 [21].^21^ Strictly speaking, what was approved by the March 2020 order was the *location* of treatment provision. Whilst the use of electronic means to carry out a consultation is mentioned, it ought to be viewed more as a procedural note to inform practitioners in simple terms how they might comply with legal requirements. This is because there was no explicit legal prohibition on the use of telemedicine in the provision of abortion care before this approval order. There were instances where remote consultations were carried out prior to the COVID-19 pandemic, albeit this was not standard practice.^22^ The reason telemedicine had not previously been embraced by abortion providers was simply that it afforded no real benefit when the patient was still required to attend a clinic in person to be prescribed and to then administer mifepristone.^23^ A patient could, legally, have undergone a remote consultation and then attended the clinic for treatment—which may have saved a patient time if it transpired that they were ineligible for treatment—but given that consultation and treatment would ordinarily have taken place during the same clinic visit, there would rarely have been a difference in convenience using telemedicine. Indeed, due to the in-person treatment requirement the use of teleconsultation would have lengthened the overall encounter for some patients.

The March 2020 approval order made two location-based changes. First, that the home of a registered medical practitioner became an approved class of place for the prescription of both mifepristone and misoprostol. Second, that the home of a pregnant person^24^ became an approved class of place for the treatment for abortion (i.e., administering both mifepristone and misoprostol per instructions from the prescribing registered medical practitioner). As such, it became lawful for both parties to remain in their respective homes for the duration of the clinical encounter.

In 2020, Christian Concern challenged the approval orders enabling TEMA on the grounds that they were *ultra vires* the powers afforded to government ministers in the AA 1967 [52]. The organisation argued that the AA 1967 was intended to ensure that abortion took place in safe and hygienic conditions, and that individuals’ homes could not meet these standards. One of their reasons for this contention was the alleged increase in ‘coerced abortions’ (abortions taking place in the absence of informed consent because an individual is subject to undue influence on the part of another person) that might occur where consultation takes place remotely (para 51) [52]. This argument, and others claiming that the home was a relatively unsafe location for abortion, ultimately failed as the Court of Appeal accepted expert evidence attesting that issues with safeguarding and consent could be appropriately identified during remote consultation [52].

As already noted, Scotland also issued a similar approval order to permit TEMA—this was issued on 31 March 2020, the same day as Wales’. There are some slight differences between the Scottish order and the English and Welsh orders which have been detailed elsewhere [50, 51]. However, given our focus on the Care Act 2014 in England we will not discuss these differences here.

### Safeguarding Concerns

The abovementioned approval was not universally welcomed. Various organisations and individuals have expressed concerns about the impact of the changes.^25^ Some were more broadly about the safety of abortion care being delivered in this way—as noted above, the safety of TEMA is well established [25]. Other concerns, and those which we will now focus on, are in relation to patient safeguarding and the question of whether this can be effectively carried out when care is not taking place face-to-face.

We will detail safeguarding duties and their statutory footing per the Care Act 2014 in the next section, but, briefly, safeguarding refers to the responsibility of (healthcare) professionals to be alert to and promote the wider welfare of their patients, ‘protecting a person’s right to live in safety, free from abuse and neglect’ (para 14.1) [18]. The purpose is to protect vulnerable individuals by identifying and appropriately responding to instances of abuse or neglect (para 14.1) [18]. Whilst the terminology of safeguarding is not universally deployed, a similar notion of promoting the wider welfare of one’s patients is observable in many countries [57].

Those who raise safeguarding concerns in relation to TEMA suggest that where a consultation takes place remotely, the ability of healthcare professionals to identify instances of, for example, coerced abortion will be compromised. Antonia Tully, Director of Campaigns for the Society for the Protection of Unborn Children, has stated that remote provision of care will result in ‘very little chance of establishing whether the woman is being coerced into abortion’ [62]. In an article advising its supporters how to respond to the public consultations on TEMA, Christian Concern raised a similar objection that ‘[t]he telemedicine system is open to abuse and deception’ because its nature is such that ‘women may not be able to speak confidentially on the call without an abuser or coercive family member hearing’, resulting in them being ‘forced into taking abortion pills’ with only their abuser present [16]. This, like the many objections raised by Christian Concern, is stated as being based on the belief that ‘[w]e must always consider the safety and wellbeing of those being served’ [16]. Another organisation that has sought to mobilise its supports in opposing TEMA—Christian Action Research and Education—raised concern about the ‘adequacy of safeguarding vulnerable clients via telemedicine’, citing increases in reports of domestic abuse during the COVID-19 pandemic as presenting further challenges to safeguarding [15].

In the House of Commons, too, these concerns have surfaced—notably in relation to the passage of the Domestic Abuse Act 2021 amidst the COVID-19 pandemic.^26^ A proposed amendment—New Clause 28—moved to amend the AA 1967 to introduce an exemption to the requirement that a patient attend a clinic for treatment in cases where the patient is unable to ‘by reason of the abusive behaviour of a person with whom the woman is personally connected’ (p. 31) [37]. At the time this new clause was tabled—July 2020—the TEMA approval orders were in force, but the amendment was intended to secure permanency for this particular vulnerable group. In tabling the new clause, Dame Diana Johnson MP highlighted the support of several organisations and abortion care providers,^27^ and explained that ‘for women in abusive relationships, who have to account for their time, their location and their spending, it can be impossible to safely travel to an abortion clinic’.^28^

However, several of her colleagues took issue with the amendment. Tim Loughton MP spoke of the potential for ‘detrimental impact’, with a risk of TEMA enabling abusers to force their partner into having an abortion ‘without the scrutiny of clinicians’.^29^ Sir Robert Neill MP raised the concern that the provider cannot know who is with the patient during a telephone consultation, summarising as follows: ‘[a]lthough the intentions are good and well meant, I have a concern about moving down the route set out in new clause 28’.^30^

Two Members spoke of correspondence they had with clinicians. One consultant, Sir Edward Leigh MP recounted, questioned ‘how there would be any confidence in detecting an abusive relationship on the basis of a telephone conversation or audio-visual interview’.^31^ Leigh himself added a worry that body language and visual signs could be missed during such consultations, and that ‘a woman seeking an abortion under duress may be being observed by abusive partners, or are otherwise acting in fear, and they will be less likely to come forward and disclose abuse’.^32^ Fiona Bruce MP summarised her position in the words of a GP who wrote to her: ‘[i]t is extraordinary that it should be argued that a woman suffering or at risk of domestic abuse, seeking abortion should somehow be considered to be at less risk if she consults a doctor remotely by telemedicine and given abortifacients to take at home’.^33^

Despite support from others in the House, this proposed new clause was ultimately dropped on the basis that the COVID-19 measures to permit TEMA would remain in place until the conclusion of the public consultation.^34^ Whilst not our focus here, it is at least worth highlighting that the public consultation to which Victoria Atkins MP referred in dropping the proposed new clause was the English one, whereas the Domestic Abuse Act 2021 extends to both England *and* Wales.^35^ It was—and, indeed, still is—possible, then, that the Welsh Government could revoke its TEMA approval before any consultation outcome, as the assurances in the House of Commons would not extend to the Welsh approval order.

Safeguarding concerns have begun to arise in responses to the public consultations on TEMA across Great Britain. Not least because organisations such as Christian Concern were actively encouraging people to oppose the change, with safeguarding concerns as a central element of the advice for responding that they provided, as already highlighted. At the time of writing, the only nation to have published any results from its consultation is Scotland. In these, many of the concerns raised around safety relate to safeguarding—confirming the patient’s identity and the safety of their home environment and identifying abuse or coercion (p. 5) [60]. It must be recognised—and the report from the Scottish Government makes this distinction in its presentation of the data—that a significant proportion of respondents submitted stock responses orchestrated by pro-life organisations (p. 4) [60].^36^ Indeed, approximately half of all responses were that publicised by Right to Life (p. 4) [60]. Where the Right to Life standardised responses are removed from the data, the positive:negative percentage split on the question of TEMA’s impact on safety shifts from 21:74 to 43:47 (p. 4) [60]. Of course, these views remain valid, but their prevalence is taken more as an indication of these organisations’ ability to mobilise supporters than as a representation of wider public opinion. Whilst results have not yet been published in England and Wales, it is fair to assume that similar objections will be raised. In England especially, as in its consultation respondents were specifically asked questions relating to objections to TEMA, including about safeguarding concerns [24]—such questions were not included in the other consultations.

There are, then, two types of safeguarding claims that are made. The first, most commonly raised, relates to the abortion treatment itself—that the treatment may be coerced, and therefore there should be some intervention to prevent this kind of occurrence from being possible. There is a concern that, for example, a patient’s abuser could be behind the camera during a video consultation, coaching the patient’s answers or otherwise intimidating them. This, it is argued, would be more easily noticed during an in-person consultation when the healthcare professional is also able to see anyone accompanying the patient. These cases do not exclusively fall under a doctor’s responsibility under the Care Act 2014, but also their more general ‘responsibility for obtaining informed consent from their patients’ (p. 64) [36]. To avoid potential criminal liability for assault or civil liability for battery doctors must only issue a prescription for abortifacients where they have the *voluntary* consent of the patient (p. 63) [36]. This particular concern, then, includes aspects of ‘safeguarding’ per common parlance that are beyond the remit of the Care Act 2014. That is not to say that they do not need addressing, but that the terminology can be slightly misleading.

Second, there are concerns related to the patient’s broader safety and wellbeing. These claims could relate to the abortion consultation as a ‘unique’ opportunity to identify adults at risk. The concern in these instances would be that some individuals may have limited contact with other institutions. For example, victims of modern slavery and sexual exploitation, whereby perpetrators have incentives to ensure that their victims do not regularly engage with any professionals. It might be thought that such perpetrators may make an exception for their victim to access abortion care, and that the requirement for an in-person consultation provides a safe space for that victim to disclose. This claim is, at its root, entirely about a doctor’s ability to perform the obligations under the Care Act 2014, specifically their obligations to identify adults who may have safeguarding needs and to act in response to any immediate risks (p. 13) [8]. For the avoidance of confusion, we will approach these claims individually.

## Satisfying the Requirements of Safeguarding

### Understanding Safeguarding

The formalised system of safeguarding that currently exists in England is relatively new, having come into effect as recently as 2015 with the implementation of the Care Act 2014. Safeguarding responsibilities existed prior to this, but the lack of clear laws meant that it had ‘been very unclear who [was] responsible for what, in practice’ [22].^37^ The purpose of the Care Act 2014, then, was to make clear various responsibilities of different groups of professionals, thereby improving safeguarding practice. As stated by the Department of Health,^38^ ‘[t]he Care Act 2014 puts the principle of individual wellbeing and professional practice of the individual [healthcare or] social worker at the heart of adult [health and] social care’ [20].

The resulting definition of safeguarding, contained in the statutory guidance,^39^ is as follows:

Safeguarding means protecting an adult’s right to live in safety, free from abuse and neglect. It is about people and organisations working together to prevent and stop both the risks and experience of abuse or neglect, while at the same time making sure that the adult’s wellbeing is promoted including, where appropriate, having regard to their views, wishes, feelings and beliefs in deciding on any action. This must recognise that adults sometimes have complex interpersonal relationships and may be ambivalent, unclear or unrealistic about their personal circumstances (para 14.7) [18].

This acknowledgement that personal circumstances are complex is indicative of the ‘[m]aking safeguarding personal’ approach advanced by the statutory guidance (para 14.15) [18], whereby the Care Act 2014 ‘sought to make adult safeguarding more outcome-focused and person-centred’ (p. 91) [35]. It is a recognition that there are various circumstances in which someone may be in need of safeguarding support, as highlighted further in the statutory guidance’s definition of abuse and neglect (which we will shortly detail).

Various duties are placed on individuals and bodies involved in the delivery of health and social care in England by the Care Act 2014. The Act itself is framed largely in terms of the responsibilities of local authorities, as they hold ultimate responsibility. However, statutory guidance from the Department of Health^40^ is clear that ‘safeguarding duties have a legal effect in relation to organisations other than the local authority on for example the NHS [National Health Service] and the Police’ (para 14.4) [18]. This can also be taken to extend to NHS arm’s-length bodies (providing NHS care under contract), which the majority of abortion providers in England are (i.e., the British Pregnancy Advisory Service, MSI Reproductive Choices, and the National Unplanned Pregnancy Advisory Service). The British Medical Association (BMA) states that ‘[s]afeguarding adults is straightforwardly part of good medical care, linked to both patient safety and overall wellbeing’ (p. 13) [8]. The BMA outlines several steps that doctors must take to discharge their safeguarding responsibilities to vulnerable adults, including: identifying adults with potential safeguarding needs; responding to any immediate risks; assessing vulnerable adults’ needs; and responding to harm or abuse by identifying relevant services who can engage (among other things) (pp. 13–15) [8].

Safeguarding duties come into play where there is suspected or reported abuse or neglect. Further to the broad person-centred approach of safeguarding, the statutory guidance provides a far-reaching definition of abuse and neglect, stipulating that professionals ‘should not limit their view of what constitutes abuse or neglect, as they can take many forms and the circumstances of the individual case should always be considered’ (para 14.17) [18]. That being said, an explicitly non-exhaustive list of examples is provided, comprising physical abuse, domestic violence, sexual abuse, psychological abuse, financial or material abuse, modern slavery, discriminatory abuse, organisational abuse, neglect and acts of omission, and self-neglect (para 14.17) [18].^41^ Of these, several may feasibly arise in the context of abortion care in an instance where a patient is seeking a termination as a result of some manner of duress. Where these instances do arise, the behaviour—meaning controlling or coercive behaviour in an intimate or family relationship—is itself a crime under Section 76 of the Serious Crime Act 2015.

GMC guidance specifies that doctors should ‘make decisions about how best to support and protect adult patients in partnership with them, and should focus on empowering patients to make decisions in their own interests [and] must support and encourage patients to be involved, as far as they want and are able, in decisions about disclosing their personal information’ (para 52) [27]. Further, if the adult lacks capacity and the doctor believes that they may be ‘experiencing, or at risk of, neglect or physical, sexual or emotional abuse, or any other kind of serious harm’, the doctor must give information about this promptly to an appropriate responsible person or authority unless they believe ‘it is not of overall benefit to the patient to do so’ (para 55) [27]. If the patient believed to be vulnerable has capacity then consent should be sought before making a disclosure, and doctors should ‘usually abide by the patient’s refusal to consent to disclosure, even if their decision leaves them (but no one else) at risk of death or serious harm’ (para 59) [27]. It is encouraged, however, that efforts are made to encourage the patient with information and support, and potentially arranging contact with support agencies [27]. The reasons for refusal of support should be recorded, and on-going support offered (p. 24) [8]. The GMC emphasises that patients may change their minds about support if they are appropriately encouraged (para 59) [27]. There are some exceptions where disclosure without consent may be appropriate. The BMA gives some examples in their safeguarding guidance including where a criminal offence has been committed, where there may be significant risk to another person, or where a suspected abusive adult is in a position of authority (p. 24) [8].

Section 42 of the Care Act 2014 details a three-part test to determine whether the local authority has a duty to make (or cause to be made) necessary enquiries into the case. Enquiries are required where the patient in question:

- Has need for care and support (whether or not the authority is meeting any of those needs);^42^
- Is experiencing, or is at risk of, abuse or neglect;^43^ and
- As a result of those needs is unable to protect himself or herself against the abuse or neglect or the risk of it.^44^

This test similarly applies for professionals to determine whether a referral to the local authority is required. Such a referral would generally be to the relevant Safeguarding Adults Board, boards which were established by local authorities in line with Section 43 of the Care Act 2014.

Where there is a safeguarding duty and suspected or reported abuse or neglect, the statutory guidance details six principles that are central to adult safeguarding:

- Empowerment: ‘People being supported and encouraged to make their own decisions and informed consent’.
- Prevention: ‘It is better to take action before harm occurs’.
- Proportionality: ‘The least intrusive response appropriate to the risk presented’.
- Protection: ‘Support and representation for those in greatest need’.
- Partnership: ‘Local solutions through services working with their communities. Communities have a part to play in preventing, detecting and reporting neglect and abuse’.
- Accountability: ‘Accountability and transparency in delivering safeguarding’ (para 14.13) [18].

The GMC emphasises empowerment, proportionality, protection, and partnership as key in the context of medical practice [28]. Taken together, these amount to ensuring that prompt action is taken where there is the belief that a patient’s ‘safety, dignity or comfort is or may be seriously compromised’, supporting patients in ‘caring for themselves to empower them to improve and maintain their health’, respecting patient confidentiality (and respecting capacitious decisions about disclosure of personal information), and listening to patients [28].

It is apparent that there may be some instances where, in the delivery of abortion care, healthcare professionals may encounter a vulnerable person who is suffering from neglect or abuse, whether that care is delivered in person or through telemedicine. As such, it is at least understandable that there is some examination of how safeguarding can be effectively carried out in the provision of TEMA—because it is (in Great Britain) a new care pathway. However, there are clear reasons, supported by the available data, to consider safeguarding suitably possible in the provision of TEMA. In what follows we discuss the two ‘types’ of safeguarding concern separately—first, we discuss instances related to abuse and neglect generally, and then we discuss the specific instance of coerced abortion, and how these instances (rare as they are) can be appropriately identified and managed during teleconsultation.

### Identifying Adults with Safeguarding Needs During Teleconsultation

Central to the safeguarding concerns raised about TEMA is the worry that a consultation carried out by telephone or video call prevents the provider from identifying—and, by extension, responding to—safeguarding concerns as effectively as they might in an in-person consultation. Providers may struggle to identify individuals who are, or are at risk of, suffering from abuse, and to take appropriate steps to aid them so they ‘receive the support they need to protect themselves from abuse and neglect’, or, where they ‘are less able to protect themselves, health professionals should take reasonable and proportionate steps to ensure their protection’ (p. 17) [8]. It is the case that fully telemedical services do not involve a face-to-face interaction, and existing sources of guidance for professionals assume this, referring to visual indicators such as physical/observable signs of abuse (e.g., unexplained injuries) to look for to identify safeguarding instances. However, that guidance has been framed in these terms does not necessarily preclude adequate safeguarding during teleconsultation.

At the outset of this discussion, it is necessary to distinguish between telephone and video consultations as they are, for various reasons, different in nature—particularly in relation to safeguarding concerns. Many objections noted earlier explicitly discuss telephone consultations as problematic. The concern is that with only *audio* communication, *visual* signs of abuse and neglect will be missed. Certainly, visual signs of distress and coercion (as well as noticeable injuries) are indicators of abuse that can be more easily observable. However, concerns focused on *telephone* consultations are not logically sound. This is because TEMA does not necessitate *telephone* consultations but can (and does) incorporate a visual element through video consultation.^45^ At most, then, this concern logically entails an objection to telephone consultations but not to TEMA in general. Nonetheless, we will demonstrate that adequate steps can be taken to identify vulnerable adults during a TEMA consultation, whether conducted by telephone or video call.

Where a consultation takes place on video call, it will still be possible to pick up on some cues that indicate abuse/coercive control. This may be important in instances where a patient’s abuser is present, because if being watched by their abuser a patient is highly unlikely to just *say* that they are being abused. It is in such instances that professionals may feel that they must rely on visual cues, such as a reluctance to make eye contact^46^ or a general timidity and attempt to keep oneself small, as means of identifying a vulnerable adult. These kinds of visual cues will still be observable during a video call. Such a medium of communication may, however, make it easier to miss injuries on some areas of the body (e.g., legs and arms). It is important to note, however, that these sorts of injuries would most likely be covered during in-person interactions. Moreover, it is possible that signs of abuse and neglect can still be identified through telephone consultation where professionals are appropriately attentive in their communication.

As the BMA notes, aspects of safeguarding centre on good communication because ‘discussion with adults can involve broaching sensitive subjects, including concerns about harm or abuse, and this requires good communication skills’ (p. 45) [8]. Good communication is a ‘basic medical skill, and many of these points are common to all discussions between doctors and patients’ (p. 44) [8]. During remote consultation, it is true that professionals can be less reliant on visual indicators when communicating. However, they can still engage in appropriate and direct conversation to ascertain the safety of the patient. As recommended by NHS England, such conversations should be appropriately framed to provide context to direct questions—for example, noting that questions about violence at home are asked routinely [47].

BPAS has developed a list of safeguarding risk assessment questions for clinicians designed to open dialogue where there are concerns, including, ‘Do you feel safe at home?’, ‘Have you ever been made to feel scared or uncomfortable by the person(s) you have been having sex with?’, ‘Do you have friends or family who you can talk to?’, and ‘Do you suffer from feeling down/depression?’ (p. 3) [46]. These questions (and others) can prompt indications of abuse where applicable, even where the questions are not directly asking about *abuse* at home. Close attention must then be paid to answers, considering signs of a ‘vague, inconsistent or implausible account of themselves and the origins of their presenting complaint’ (p. 56) [8] and audio clues such as changes in tone or strength of voice, or a general lack of certainty and diffidence in responses. Paperwork can also be used to identify potential vulnerable people. For example, individuals who are not registered with a GP or who have moved frequently may be potential victims of trafficking. At most, the concern about a lack of visual clues in remote consultation might suggest a need for more specific training—it is true that healthcare professionals are largely trained for in-person patient contact and may need assistance in adjusting to carrying out consultations by telephone or video call.

In addition to adequate safeguarding conversations being *possible* by telephone/video call, these encounters are actually *more likely* where consultation takes place remotely. Prior to the approval of TEMA, one of the most common reasons given for accessing abortion medications unlawfully was that abuse at home prevented individuals from attending a clinic—either because the person’s movements were closely controlled by an abuser, or out of fear that an abuser would find out they were pregnant/seeking abortion [2]. Other vulnerable people—such as victims of trafficking—are rarely allowed to engage with formal health services. The reality is, therefore, that ‘[t]he absence of remote care is […] forcing these women into a situation entirely devoid of safeguarding. Even if in-person care was considered to be the ideal, remote provision results in fewer women having no access to care or accessing care unlawfully’ (p. 557) [57]. Remote consultation increases the likelihood of vulnerable adults being identified because they may choose formal channels where they can access them without attending a clinic and administer treatment at home when the abuser is out, or in a regularly attended space outside the home without raising suspicion. Some people may still fall through the net and not be identified as vulnerable, but this happens when care is provided in person. It is better that these individuals at least have some institutional contact that otherwise is unlikely to happen, as it can enhance their trust in healthcare services and potentially enable them to disclose in future.

Further, regardless of their ability to attend a clinic in person, an individual may feel more comfortable making a disclosure during a remote consultation. One might argue that a remote consultation interferes with the empowerment principle. An in-person consultation might be said to enable better rapport and put the patient at ease, which may make them more likely to disclose their situation. This is, however, reflective of neither communication in the modern world nor the individuality of patients’ healthcare preferences. The COVID-19 pandemic has quite clearly demonstrated that a lot can be achieved using telephone or video calls where in-person interaction would previously have been the standard. We are in an age where people are very used to online communication. Many people are actually more comfortable with this, and there is no reason why rapport cannot be built between patient and provider during a remote consultation. Further, many people do not feel at ease in clinical environments. The British Pregnancy Advisory Service has reported that feedback from patients ‘suggests women find it easier to disclose concerns via telephone in their own homes (or location convenient to them) as opposed to in a clinical environment’ (p. 4) [46]. This could be because they feel better empowered in a familiar environment, free of some of the intimidating aspects of a clinic or hospital (p. 556) [57]. This is particularly true for population groups who have historically found interacting with healthcare professionals more difficult. For example, women and ethnic minorities often find that they are misdiagnosed, or that they are treated as unbelievable narrators of their own symptoms [6]. A clinic may not, then, be the safe haven for victims of abuse that it is sometimes portrayed as, and TEMA may better satisfy the empowerment principle for some vulnerable patients.

The statutory guidance requires professionals to ‘look beyond single incidents […] to identify patterns of harm’ (para 14.18) [18]. This is something that is not ordinarily a possibility in abortion care because patients can self-refer to abortion providers and, as of the approval of home use of misoprostol in 2018, accessing EMA has necessitated only one consultation.^47^ Even before that, only two consultations were ordinarily required. There is not, then, the continuity of care that is customary in primary care or the care of a patient with a chronic condition. However, this has not changed with the introduction of TEMA. The number of consultations in the care pathway has remained the same, and it is simply the medium used that has changed. As such, it cannot be said that TEMA has *reduced* the ability of abortion providers to execute their safeguarding duty. One could argue that the move to requiring only one consultation had this effect, but such an argument would ultimately be unconvincing given the number of clinical encounters that are standalone; an accident and emergency (A&E) admission, for example, presents a single clinical encounter (assuming no follow-up is required), and it is just as possible that a patient attending A&E is the victim of abuse or neglect (that abuse or neglect potentially having been the cause of their A&E attendance).

### Ensuring that There is no Coercion During Teleconsultation

In this section, we address the specific concern that has been raised about TEMA enabling coerced abortion [62]. This concern arises from the fact that where a consultation is not in person it cannot be verified that the patient is alone during the consultation. Healthcare professionals are advised to ‘consider the environment’ when undertaking safeguarding, including not asking direct questions about safety at home in the presence of family members or friends [47], which may be considered challenging during a remote consultation. Equally, it may be thought difficult to ensure the patient’s consent to treatment is voluntary where there is another person present, especially if the healthcare professional cannot see how that other person is behaving around the patient—for example, they may be coaching responses to questions or threatening the patient. Opponents to TEMA suggest that an in-person appointment enables the provider to see the patient alone, thereby providing an opportunity for them to disclose abuse, and for the clinician to be confident that the patient is seeking abortion of their own volition [16].

Again, this fails to suitably distinguish between in-person care and TEMA. In seeking to keep hidden their abuse, an abuser may well attempt to attend the clinic with their victim to prevent them confiding in someone. Of course, if the provider had reason to suspect a safeguarding matter, they could request to speak to the patient alone. However, even if the abuser is not present in the consulting room, the patient’s fear of the repercussions of disclosing can still impact on their ability to raise concerns about, or answer questions about, their safety honestly (p. 556) [57]. As noted above, the likelihood of a patient having a consultation without their abuser being aware or present is higher with TEMA because it is easier to make a secret telephone or video call than it is to account for whereabouts when attending a clinic. In fact, we can see from the jurisprudence around forced abortion (which is a crime [36]^48^) that abusers source the medications unlawfully because this reduces the chance of them being caught. In instances of forced abortion, the unfortunate reality is that many abusers will continue to take this approach.

Where abusers do allow their pregnant victims to engage with formal healthcare services and attempt to coach their responses through the consultation, this could be noticeable. For example, the patient’s eyes may keep glancing over the screen during a video call, or there may be long pauses/voices in the background during a telephone call. The provider can first ask additional questions in seeking to satisfy themselves that there is no cause for concern and, if that is insufficient, exercise clinical discretion in insisting on an in-person consultation. Exercising such discretion is squarely within the principles of safeguarding, seeking to prevent harm and protect the suspected victim, and doing so in a proportionate manner.

Even if safeguarding concerns are raised and ‘an adult is regarded ‘at risk’ [this] is not by itself evidence that capacity is lacking, and care must be taken to avoid any assumption’ (p. 24) [8]. Just because a person is a victim of abuse, does not mean that their consent to abortion will be involuntary. There will be victims of abuse that are being forced to continue a pregnancy that they do not want who would be prevented from visiting a clinic in person by their abuser(s), but whom may be able to find sufficient time and privacy to access TEMA. For these individuals, the ability to access this care is a matter of safety, both because continuing the pregnancy can increase the abuser’s power over them (many report feeling ‘trapped’ because of the pregnancy) [5] and because violence is known to escalate during pregnancy [11, 17].

The focus of much of the discourse around TEMA—especially that of the organisations opposed to it that we have already discussed—is on people who may be pressured into abortions and how they can best be protected, rather than those victims of abuse who need abortions. This demonstrates the political motives underpinning such objections. It is no secret that the key organisations that are vocally opposing TEMA are distinctly anti-abortion, often on the basis of religion. An organisation truly concerned with safeguarding pregnant people who are accessing (or want to access) abortion services would duly acknowledge that there are two sides and that TEMA affects them in different ways.

## Conclusion

Concerns about safeguarding arising with the introduction of TEMA were inevitable. The general shift in the direction of telemedicine during the COVID-19 pandemic caused many to question the negative impact this could have on vulnerable people, and this naturally extends to those accessing abortion care. However, there are strong reasons—and a growing evidence base—to suggest that such concerns are misplaced in the context of TEMA. We have demonstrated this in relation to two key concerns raised by opponents of TEMA: (1) that a remote consultation prevents a healthcare professional from identifying instances of abuse, and (2) that healthcare professionals cannot reliably confirm the absence of coercion during a remote consultation.

Ultimately, there is no reason why safeguarding cannot be carried out during the provision of TEMA to the same standard as during the provision of in-person EMA care. There is even evidence to suggest that safeguarding efforts may be improved where TEMA is available, as victims of abuse may find it easier to access such care without their abuser knowing—it is easier to find time alone for a remote consultation than to account for one’s whereabouts when attending an in-person appointment. Whilst some individuals in need of a safeguarding intervention will inevitably fall through the net, this is inherently unavoidable, and only permitting the provision of EMA in person at best does nothing to prevent this, and at worst increases the chances.

The safeguarding concerns raised about TEMA can, then, be set aside. Doing so removes the most significant objection to the present (temporary) approval order in England—and, indeed, those in Scotland and Wales—thereby strengthening the case for a permanent change to the law.

## Footnotes

1 Section 3(a) of the Abortion Act 1967, as inserted by the Human Fertilisation and Embryology Act 1990, extends the powers of the respective health secretaries to approve a ‘class of places’ for the purposes of abortion treatment. For further discussion of these powers see [56].

2 Incidentally, this response to enable the continuation of abortion care services was not universal [54].

3 Similar legislation exists in both Scotland and Wales by way of the Adult Support and Protection (Scotland) Act 2007 and Social Services and Well-being (Wales) Act 2014, respectively.

4 Section 58, Offences Against the Person Act 1861.

5 Section 1(2), Infant Life (Preservation) Act 1929.

6 Section 5, Abortion Act 1967.

7 Section 1(1)(a), Abortion Act 1967.

8 Section 1(1)(a), Abortion Act 1967.

9 Section 1(3), Abortion Act 1967.

10 Section 1(3A), Abortion Act 1967. See the limits outlined in the approval orders discussed later in the paper.

11 We specify fully because, as we will come to discuss, telemedicine could have been used for *some* elements of the care pathway.

12 Women on Web has been providing TEMA since 2004, but only to those in countries where access to abortion is legally restricted.

13 If the patient’s nearest clinic has an on-site doctor, then the consultation will take place in person. The TEMA service was introduced to enable more of the affiliate’s clinics to offer the service.

14 The prescription is provided by the off-site doctor remotely unlocking a drawer in the consultation room containing both mifepristone and misoprostol.

15 A largely similar service—the TeleAborto Study—has been operated in Mexico by the same organisation since 2019. Further information about the study can be found on its website [64]. A similar model was also launched earlier, in 2015, by the Tabbot Foundation in Australia [38].

16 At the time of writing, TelAbortion operates in Colorado, Georgia, Hawaii, Illinois, Iowa, Maine, Maryland & Washington D.C., Massachusetts, Minnesota, Montana, Nevada, New Jersey, New Mexico, New York, Oregon, Virginia, and Washington.

17 Estimating gestation by last menstrual period has been found to be effective [59]. As such, the World Health Organization recommends self-assessment of eligibility for medical abortion—though also highlights a need for further research to clarify some specific uncertainties [68].

18 Such benefits include contributing to the overcoming of health inequities, improve the efficiency of services, and enhancing the autonomy of patients [49].

19 By this we mean either where there is an indication for an in-person consultation prior to the procedure (i.e., risk of ectopic pregnancy), or a reason for in-person care in the event of complications (i.e., haemorrhaging). This is in line with the model operated by the British Pregnancy Advisory Service, as described above [9].

20 Among these studies were those analysing the Women on Web service, as mentioned above.

21 Incidentally, a near identical approval order was issued in England a week earlier, on 23 March 2020, but was removed from the Department of Health and Social Care website within hours on the grounds that it had been an ‘error’. See [55].

22 This is based on personal communication with care providers.

23 Prior to the approval order a few years earlier permitting home use of misoprostol, patients ordinarily had to attend the clinic twice to take each drug separately.

24 The approval order states ‘pregnant woman’.

25 Some of the concerns we highlight here were raised in relation to the Scottish or Welsh approvals, but can be taken as applicable to that in England.

26 The Domestic Abuse Bill had its first reading on 3 March 2020 and had been in the works long before the COVID-19 pandemic. It was not, therefore, in response to the impact of the COVID-19 pandemic on domestic abuse. However, the timing was such that its journey through Parliament was almost entirely within pandemic restrictions.

27 There was support, noted Johnson, from the Royal College of Obstetricians and Gynaecologists, the Royal College of Midwives, the Faculty of Sexual and Reproductive Healthcare, the British Society of Abortion Care Providers, the British Pregnancy Advisory Service, Marie Stopes, the End Violence Against Women Coalition, and Women’s Aid.

28 HC Deb 6 July 2020, vol 678, col 720.

29 HC Deb 6 July 2020, vol 678, cols 722–723.

30 HC Deb 6 July 2020, vol 678, col 719.

31 HC Deb 6 July 2020, vol 678, col 732.

32 HC Deb 6 July 2020, vol 678, col 733.

33 HC Deb 6 July 2020, vol 678, cols 735–736.

34 HC Deb 6 July 2020, vol 678, col 769.

35 Section 89(1), Domestic Abuse Act 2021.

36 Of course, similar campaigns using standardised responses are run by pro-choice organisations. However, they are less prevalent—to the point that analyses are not provided in an additional format to exclude them.

37 Indeed, this lack of clarity resulted in the severe cases of neglect at Southern Cross’ Orchid View care home, as came to light in 2014 [12].

38 Now Department of Health and Social Care.

39 This guidance replaced the Department of Health’s (now Department of Health and Social Care) previous *No secrets* guidance [19].

40 Now Department of Health and Social Care.

41 Given the broader applicability of safeguarding duties—i.e., beyond local authorities—these categories are also listed in the General Medical Council’s website resource on safeguarding [28].

42 Section 42(1)(a), Care Act 2014.

43 Section 42(1)(b), Care Act 2014.

44 Section 42(1)(c), Care Act 2014.

45 Note that as a matter of policy, the British Pregnancy Advisory Service requires all patients under 18 to be consulted by video call. See [46].

46 In the case of a video consultation, the equivalent would be a reluctance to look directly ahead at the camera/screen.

47 Home use of misoprostol was approved in Wales and England in 2018. It was approved in 2017 in Scotland. See [48]

48 Section 58, Offences Against the Person Act 1861.
